# Correlation between myocardial work and disease activity in rheumatoid arthritis patients with preserved left ventricular ejection fraction: a retrospective study based on non-invasive pressure-strain loop左心室射血分数保留的类风湿性关节炎患者心肌功与疾病活动度的相关性：一项基于无创压力-应变回路的回顾性研究

**DOI:** 10.1007/s10067-025-07380-5

**Published:** 2025-03-05

**Authors:** Xiaolong Yu, Jing Xi, Jiabiao Wu, Ruixiao Song

**Affiliations:** 1 Department of Ultrasonics, Wujin Hospital Affiliated with Jiangsu University, Changzhou, Jiangsu China; 2 Rheumatology and Immunology Department, Wujin Hospital Affiliated with Jiangsu University, Changzhou, Jiangsu China

**Keywords:** Left ventricular pressure-strain loop, Myocardial work, Rheumatoid arthritis, Speckle-tracking echocardiography

## Abstract

**Background:**

Early cardiac damage is very common in RA patients, but it is usually subclinical. Therefore, finding a non-invasive method for the early detection and treatment of cardiac damage in autoimmune diseases is particularly important.早期diac dam年龄在 RA 患者中很常见，但通常是亚临床的。因此，寻找一种非侵入性方法来早期检测和治疗自身免疫性疾病中的心脏损伤尤为重要。

**Objective:**

To evaluate left ventricular function changes in rheumatoid arthritis (RA) patients with preserved left ventricular ejection fraction (LVEF) using left ventricular pressure-strain loop (LV-PSL) technology and to explore the correlation between myocardial work (MW) and disease activity.使用左心室压力-应变环 （LV-PSL） 技术评估左心室射血分数 （LVEF） 保留的类风湿性关节炎 （RA） 患者的左心室功能变化，并探讨心肌功 （MW） 与疾病活动度之间的相关性。

**Methods:**

A total of 62 RA patients with preserved LVEF, treated at Wujin Hospital Affiliated with Jiangsu University from January 2021 to September 2023, were included. Patients were categorized into low (25), medium (18), and high (19) disease activity groups based on the 28 joint disease activity score (DAS28). A control group of 29 healthy individuals was also established. LV-PSL technology assessed left ventricular global longitudinal strain (GLS) and MW parameters: global constructive work (GCW), global wasted work (GWW), global work index (GWI), and global work efficiency (GWE). Correlations between MW parameters, GLS, LVEF, and DAS28 scores were analyzed.共纳入 2021 年 1 月至 2023年9月在江苏大学附属武进医院治疗的 62 例 LVEF 保留的 RA 患者。根据 28 项关节疾病活动评分 （DAS28） 将患者分为低 （25） 、中 （18） 和高 （19） 疾病活动组。还建立了一个由 29 名健康个体组成的对照组。LV-PSL 技术评估左心室整体纵向应变 （GLS） 和 MW 参数：整体建设性工作 （GCW）、整体浪费工作 （GWW）、整体工作指数 （GWI） 和整体工作效率 （GWE）。分析 MW 参数、 GLS 、 LVEF 和 DAS28 评分之间的相关性。

**Results:**

There were no significant differences in general data between study and control groups (*p* > 0.05). However, laboratory indicators (RF, CRP, ESR) showed significant differences (*p* < 0.05). GWI, GCW, GWE, and GLS were significantly lower in the high disease activity group compared to controls (*p* < 0.05). GWI, GCW, and GWE were positively correlated with LVEF and absolute GLS, while GWW correlated negatively with LVEF (*p* < 0.05).研究组和对照组之间的一般数据没有显著差异 （*p* > 0.05）。然而，实验室指标 （RF、CRP、ESR） 显示显着差异 （*p* < 0.05）。与对照组相比，高疾病活动组的 GWI、GCW、GWE 和 GLS 显着降低 （*p* < 0.05）。GWI、GCW 和 GWE 与 LVEF 和绝对 GLS 呈正相关，而 GWW 与 LVEF 呈负相关 （*p* < 0.05）。研究组和对照组之间的一般数据没有显著差异 （*p* > 0.05）。然而，实验室指标 （RF、CRP、ESR） 显示显着差异 （*p* < 0.05）。与对照组相比，高疾病活动组的 GWI、GCW、GWE 和 GLS 显着降低 （*p* < 0.05）。GWI、GCW 和 GWE 与 LVEF 和绝对 GLS 呈正相关，而 GWW 与 LVEF 呈负相关 （*p* < 0.05）。

**Conclusion:**

RA disease activity is closely associated with impaired myocardial work. LV-PSL technology effectively monitors myocardial function abnormalities in RA patients, providing valuable insights for clinical management.

**Table:** 

**Key Points** • *Myocardial work is significantly impaired in RA patients with high disease activity.* • *Left ventricular pressure-strain loop (LV-PSL) technology effectively assesses cardiac function in this patient population.* • *Increased disease activity correlates with reduced myocardial work parameters*.

## Introduction

Rheumatoid arthritis (RA) is a systemic autoimmune disease that increasingly raises concerns among clinicians regarding extra-articular manifestations. The cardiac is one of the common target organs affected by RA, with involvement of the pericardium, vasculature, myocardium, and valves. A 10-year matched cohort study found that patients with RA were 1.43 times more likely to develop acute coronary syndrome (ACS) than the control group (hazard ratio (HR) 1.43, 95% CI 1.10–1.84). According to the guidelines of the European Society of Cardiology, RA is considered to be an important risk factor for cardiovascular disease (CVD), and its risk of CVD is up to twice that of the general population [1]. The risk of cardiovascular death increases by up to 50% [2].

Early cardiac damage is very common in RA patients, but it is usually subclinical. Due to their insidious nature or atypical features, early cardiac damage in RA is frequently overlooked. When there are obvious symptoms, the cardiac damage is often serious and irreversible, putting the patient’s life at risk. Therefore, the early detection and treatment of cardiac damage in autoimmune diseases are particularly important.早期心脏损伤在 RA 患者中很常见，但通常是亚临床的。由于其隐匿性或非典型特征，RA 的早期心脏损伤经常被忽视。当有明显症状时，心脏损伤往往是严重且不可逆的，使患者的生命处于危险之中。因此，自身免疫性疾病中心脏损伤的早期发现和治疗尤为重要。

LVEF is still the preferred index for echocardiography to evaluate cardiac systolic function; however, LVEF has great limitations in the evaluation of systolic function [3]. In recent years, cardiac ultrasound imaging technology has made significant progress in the field of myocardial function assessment. In particular, non-invasive LV-PSL technology has shown its unique value in evaluating patients with preserved LVEF. This technology adds non-invasive measurement of arterial pressure on the basis of two-dimensional speckle-tracking echocardiography (2D-STE) hierarchical strain technology, which effectively mitigates the afterload influence, and provides a new dimension for evaluating myocardial work (MW). The latest research shows that parameters such as GWE and GWI are of great significance in evaluating myocardial work [4]. Meucci et al.’s [5] study showed that non-invasive left ventricular myocardial work is a new parameter to evaluate the myocardial work of patients with chronic aortic insufficiency and preserved LVEF, which can better assist clinicians in understanding patients’ myocardial function and energetics.LVEF 仍然是超声心动图评估心脏收缩功能的首选指标;然而，LVEF 在评估收缩功能方面有很大的局限性 []。近年来，心脏超声成像技术在心肌功能评估领域取得了重大进展。特别是，无创 LV-PSL 技术在评估 LVEF 保留患者方面显示出其独特的价值。该技术在二维斑点追踪超声心动图 （2D-STE） 分层应变技术的基础上增加了动脉压的无创测量，有效减轻了后负荷影响，为评估心肌功 （MW） 提供了新的维度。最新研究表明，GWE和GWI等参数在评估心肌工作中具有重要意义[]。Meucci 等 [] 的研究表明，无创左心室心肌工作是评价慢性主动脉瓣关闭不全和 LVEF 保留患者心肌工作的新参数，可以更好地帮助临床医生了解患者的心肌功能和能量。LVEF 仍然是超声心动图评估心脏收缩功能的首选指标;然而，LVEF 在评估收缩功能方面有很大的局限性 []。近年来，心脏超声成像技术在心肌功能评估领域取得了重大进展。特别是，无创 LV-PSL 技术在评估 LVEF 保留患者方面显示出其独特的价值。该技术在二维斑点追踪超声心动图 （2D-STE） 分层应变技术的基础上增加了动脉压的无创测量，有效减轻了后负荷影响，为评估心肌功 （MW） 提供了新的维度。最新研究表明，GWE和GWI等参数在评估心肌工作中具有重要意义[]。Meucci 等 [] 的研究表明，无创左心室心肌工作是评价慢性主动脉瓣关闭不全和 LVEF 保留患者心肌工作的新参数，可以更好地帮助临床医生了解患者的心肌功能和能量。

This study aims to use the advanced non-invasive LV-PSL technology to evaluate the myocardial work of RA patients with preserved LVEF. By analyzing the relationship between myocardial work and disease activity in patients, we hope to provide more individualized and precise cardiac assessments for this specific patient population. This approach seeks to identify cardiac dysfunction in patients with RA at an early stage, thus offering new insights and decision-making support for the management of cardiac function in RA patients.本研究旨在使用先进的无创 LV-PSL 技术来评估 LVEF 保留的 RA 患者的心肌工作。通过分析患者心肌工作与疾病活动之间的关系，我们希望为这一特定患者群体提供更个体化和精确的心脏评估。该方法旨在早期识别 RA 患者的心功能不全，从而为 RA 患者的心功能管理提供新的见解和决策支持。

## Data and methods

### Study design

A total of 62 patients with preserved LVEF treated at Wujin Hospital Affiliated with Jiangsu University from January 2021 to September 2023 were selected. According to the 28-joint Disease Activity Scores (DAS28) [6], the patients were divided into three groups: low disease activity group (2.6 ≤ DAS28 ≤ 3.2), 25 cases (5 males, 20 females), aged 36–77 years (median age, 58 years (51, 71.5)); moderate disease activity group (3.2 < DAS28 ≤ 5.1), 18 cases (6 males, 12 females), aged 36–72 years (median age, 63 years (53, 71)); and high disease activity group (DAS28 > 5.1), 19 cases (3 males, 16 females), aged 46–80 years (median age, 62.5 years (53.25, 72.5)). A control group consisting of 29 healthy individuals was included (10 males, 19 females; aged 24–88 years (median age, 64 years (57, 72))). Inclusion criteria are as follows: (1) sinus rhythm, (2) patients diagnosed with RA according to the 2010 ACR/EULAR classification criteria [7], and (3) conventional ultrasound showed no obvious ventricular walls dyskinesia and LVEF ≥ 50%. Exclusion criteria are as follows: (1) arrhythmia; (2) patients with left ventricular hypertrophy caused by previous hypertension, as well as patients with severe valvular disease, cardiomyopathy, congenital heart disease, and heart failure; (3) patients with renal function impairment and other connective tissue diseases; and (4) patients with poor acoustic windows on transthoracic echocardiography. Diabetes was diagnosed based on the 1999 WHO diagnostic criteria as a fasting blood glucose level ≥ 7.0 mmol/L or 2-h postprandial blood glucose (PG) level ≥ 11.1 mmol/L using the oral glucose tolerance test (OGTT). The diagnosis of hypertension was based on the ISH2020 international hypertension practice guidelines [8], requiring a systolic blood pressure ≥ 140 mmHg and/or diastolic blood pressure ≥ 90 mmHg. Smoking was defined according to the WHO definition as patients who smoked more than one cigarette per day for 6 months or longer. This study was approved by the ethics committee of our hospital. All subjects provided written informed consent prior to the examination.选取 2021 年 1 月至 2023年9月在江苏大学附属武进医院治疗的 62 例 LVEF 保留患者。根据 28 个关节疾病活动评分 （DAS28） []，将患者分为三组：低疾病活动度组 （2.6 ≤ DAS28 ≤ 3.2）、25 例 （5 例男性，20 例女性），年龄 36-77 岁 （中位年龄 58 岁 （51， 71.5））;中度疾病活动组 （3.2 < DAS28 ≤ 5.1），18 例 （6 例男性，12 例女性），年龄 36-72 岁 （中位年龄 63 岁 （53， 71））;高度疾病活动组 （DAS28 > 5.1），19 例 （3 例男性，16 例女性），年龄 46-80 岁 （中位年龄 62.5 岁 （53.25， 72.5））。纳入由 29 名健康个体组成的对照组 （10 名男性，19 名女性;年龄 24-88 岁 （中位年龄 64 岁 （57， 72）））。I排除标准如下：（1） 窦性心律，（2） 根据 2010 年 ACR/EULAR 分类标准 [] 诊断为 RA 的患者，以及 （3） 常规超声显示无明显的心室壁运动障碍，LVEF ≥ 50%。E排除标准如下：（1） 心律失常;（2） 既往高血压引起的左心室肥厚患者，以及严重瓣膜病、心肌病、先天性心脏病和心力衰竭患者;（3） 肾功能损害和其他结缔组织病患者;（4） 经胸超声心动图声窗较差的患者。根据 1999 年 WHO 诊断标准，使用口服葡萄糖耐量试验 （OGTT） 将空腹血糖水平≥ 7.0 mmol/L 或餐后 2 小时血糖 （PG） 水平≥ 11.1 mmol/L 诊断为糖尿病。 高血压的诊断基于ISH2020国际高血压实践指南[]，要求收缩压≥140mmHg和/或舒张压≥90mmHg。根据 WHO 定义，吸烟定义为每天吸烟超过一支香烟 6 个月或更长时间的患者。本研究经我院伦理委员会批准。所有受试者在检查前均提供书面知情同意书。选取 2021 年 1 月至 2023年9月在江苏大学附属武进医院治疗的 62 例 LVEF 保留患者。根据 28 个关节疾病活动评分 （DAS28） []，将患者分为三组：低疾病活动度组 （2.6 ≤ DAS28 ≤ 3.2）、25 例 （5 例男性，20 例女性），年龄 36-77 岁 （中位年龄 58 岁 （51， 71.5））;中度疾病活动组 （3.2 < DAS28 ≤ 5.1），18 例 （6 例男性，12 例女性），年龄 36-72 岁 （中位年龄 63 岁 （53， 71））;高度疾病活动组 （DAS28 > 5.1），19 例 （3 例男性，16 例女性），年龄 46-80 岁 （中位年龄 62.5 岁 （53.25， 72.5））。纳入由 29 名健康个体组成的对照组 （10 名男性，19 名女性;年龄 24-88 岁 （中位年龄 64 岁 （57， 72）））。I排除标准如下：（1） 窦性心律，（2） 根据 2010 年 ACR/EULAR 分类标准 [] 诊断为 RA 的患者，以及 （3） 常规超声显示无明显的心室壁运动障碍，LVEF ≥ 50%。E排除标准如下：（1） 心律失常;（2） 既往高血压引起的左心室肥厚患者，以及严重瓣膜病、心肌病、先天性心脏病和心力衰竭患者;（3） 肾功能损害和其他结缔组织病患者;（4） 经胸超声心动图声窗较差的患者。根据 1999 年 WHO 诊断标准，使用口服葡萄糖耐量试验 （OGTT） 将空腹血糖水平≥ 7.0 mmol/L 或餐后 2 小时血糖 （PG） 水平≥ 11.1 mmol/L 诊断为糖尿病。 高血压的诊断基于ISH2020国际高血压实践指南[]，要求收缩压≥140mmHg和/或舒张压≥90mmHg。根据 WHO 定义，吸烟定义为每天吸烟超过一支香烟 6 个月或更长时间的患者。本研究经我院伦理委员会批准。所有受试者在检查前均提供书面知情同意书。

### Instruments and methods

#### Instrument

The GE Vivid e95 ultrasonic diagnostic instrument with an M5Sc-D probe operating at a frequency of 1.7–3.3 MHz was used.

#### Image acquisition

Blood pressure was measured in a resting state 10 min before the ultrasound examination. Participants were instructed to lie in the left lateral position and connected to an electrocardiogram (ECG) machine to ensure stable ECG curves. Following the American College of Cardiology (ACC) guidelines [9], the left ventricular long-axis view from the parasternal region was obtained to measure the left ventricular end-diastolic diameter (LVEDd), interventricular septum thickness at end-diastole (IVSD), and left ventricular posterior wall thickness at end-diastole (LVPWD). The two-dimensional double-plane Simpson method was used to calculate the end-diastolic volume of the left ventricle (LVEDV), end-systolic volume of the left ventricle (LVESV), and LVEF. Participants were instructed to hold their breath while dynamic two-dimensional images were collected from the apical four-chamber, two-chamber, and long-axis views, ensuring at least three consecutive stable and clear heart cycle images were obtained. The apical long-axis view displayed clear images of the mitral and aortic valves, and Doppler spectrum of the aortic valve was performed to define the end-systolic phase. The dynamic images and still images were stored for further analysis, with a frame rate during image acquisition of 40–80 frames per second.超声检查前 10 min 在静息状态下测量血压。参与者被指示以左侧卧位躺下并连接到心电图 （ECG） 机器以确保稳定的心电图曲线。根据美国心脏病学会 （American College of Cardiology， ACC） 指南 []，从胸骨旁区域获得左心室长轴视图，以测量左心室舒张末期直径 （LVEDd）、舒张末期室间隔厚度 （IVSD） 和舒张末期左心室后壁厚度 （LVPWD）。采用二维双平面 Simpson 方法计算左心室舒张末期容积 （LVEDV） 、左心室收缩末期容积 （LVESV） 和 LVEF。指示参与者屏住呼吸，同时从根尖四腔、双腔和长轴视图收集动态二维图像，确保获得至少三个连续稳定清晰的心周期图像。根尖长轴视图显示二尖瓣和主动脉瓣的清晰图像，并进行主动脉瓣多普勒频谱以确定收缩末期。动态图像和静止图像被存储起来以供进一步分析，图像采集期间的帧速率为每秒 40-80 帧。超声检查前 10 min 在静息状态下测量血压。参与者被指示以左侧卧位躺下并连接到心电图 （ECG） 机器以确保稳定的心电图曲线。根据美国心脏病学会 （American College of Cardiology， ACC） 指南 []，从胸骨旁区域获得左心室长轴视图，以测量左心室舒张末期直径 （LVEDd）、舒张末期室间隔厚度 （IVSD） 和舒张末期左心室后壁厚度 （LVPWD）。采用二维双平面 Simpson 方法计算左心室舒张末期容积 （LVEDV） 、左心室收缩末期容积 （LVESV） 和 LVEF。指示参与者屏住呼吸，同时从根尖四腔、双腔和长轴视图收集动态二维图像，确保获得至少三个连续稳定清晰的心周期图像。根尖长轴视图显示二尖瓣和主动脉瓣的清晰图像，并进行主动脉瓣多普勒频谱以确定收缩末期。动态图像和静止图像被存储起来以供进一步分析，图像采集期间的帧速率为每秒 40-80 帧。

#### Image analysis

**Fig. 1 图 1:**
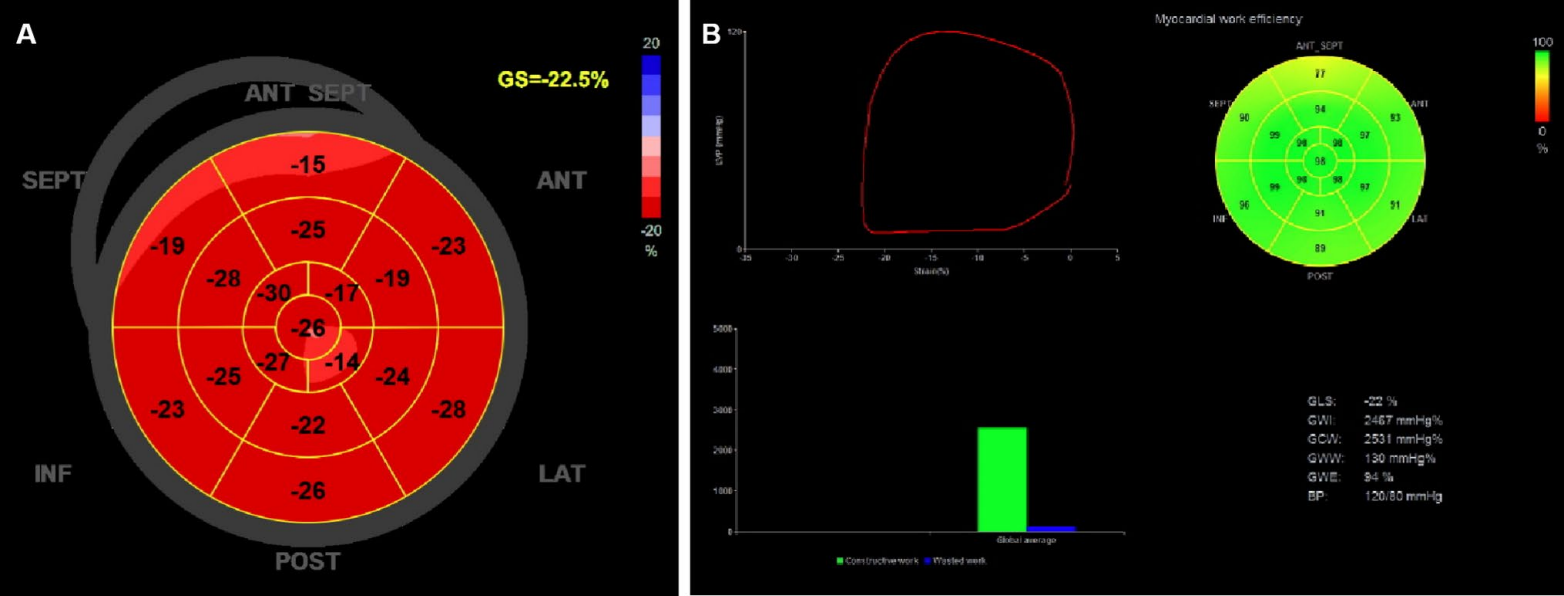
Assessment of left ventricular myocardial work using pressure-strain loop. **A** Left ventricular systolic longitudinal strain and bull’s-eye diagram of 17-segment. **B** The top left of the figure shows the non-invasive PSL loop with strain on the *x*-axis and left intraventricular pressure on the *y*-axis; the lower left shows the bars of GCW versus GWW; the upper right is the bull’s-eye diagram of 17-segment myocardial work index; the lower right shows the specific values of the myocardial work parameters. GWI, global work index; GCW, global constructive work; GWW, global wasted work; GWE, global work efficiency; EF, ejection fraction; GLS, global longitudinal strain使用压力-应变环评估左心室心肌功。**A** 左心室收缩纵向应变和 17 节段的牛眼图。**B** 图左上角显示 *x* 轴有应变、y 轴有左心室内压的无创 PSL 环;左下角显示了 GCW 与 GWW 的条形;右上是 17 段心肌功指标的靶心图;右下角显示心肌功参数的具体值。GWI，全球工作指数;GCW，全球建设性工作;GWW，全球浪费的工作;GWE，全球工作效率;EF，射血分数;GLS， 整体纵向应变使用压力-应变环评估左心室心肌功。**A** 左心室收缩纵向应变和 17 节段的牛眼图。**B** 图左上角显示 *x* 轴有应变、y 轴有左心室内压的无创 PSL 环;左下角显示了 GCW 与 GWW 的条形;右上是 17 段心肌功指标的靶心图;右下角显示心肌功参数的具体值。GWI，全球工作指数;GCW，全球建设性工作;GWW，全球浪费的工作;GWE，全球工作效率;EF，射血分数;GLS， 整体纵向应变

Blood pressure values were entered into the EchoPAC workstation, marking valve opening and closing times on the spectra obtained from the aortic and mitral valves. The automatic functional imaging mode was selected, and the endocardial borders of the apical three-chamber, four-chamber, and two-chamber dynamic images were traced. Manual adjustments were made as required to the automatically traced endocardial contours. The 17-segment model was used, and the software calculated the GLS as the weighted average of the peak systolic longitudinal strain of all segments, generating longitudinal strain curves and bull’s-eye plots for storage. If any one or more areas of the segment tracking are unsatisfactory, the participant would be excluded from the study. Then, open Event Timing to determine the opening and closing times of the aortic and mitral valves based on spectral Doppler. Input the blood pressure measured before the echocardiography examination. The “myocardiacwork” program was selected to display the bull’s-eye diagram of overall myocardial work. The overall myocardial work index (MWI) parameters of the left ventricular myocardium were obtained, which include (1) GCW, representing the positive work done by the shortening of the systolic myocardium or the prolongation of the diastolic myocardium, contributing to left ventricular ejection; (2) GWW, representing the negative work of systolic myocardial prolongation or diastolic myocardial shortening, working against left ventricular ejection; (3) GWE calculated as GCW/(GCW + GWW); and (4) GWI, which was the weighted average of the MWI of the 17 myocardial segments (see Fig. 1).将血压值输入到 EchoPAC 工作站中，在从主动脉瓣和二尖瓣获得的光谱上标记瓣膜的打开和关闭时间。选择自动功能成像模式，追踪根尖三腔、四腔、两腔动态图像的心内膜边界。根据需要对自动追踪的心内膜轮廓进行手动调整。使用 17 段模型，软件将 GLS 计算为所有段的峰值收缩纵向应变的加权平均值，生成纵向应变曲线和靶心图以供存储。如果区段跟踪的任何一个或多个区域不令人满意，则参与者将被排除在研究之外。然后，打开 Event Timing（事件计时）以确定基于频谱多普勒的主动脉瓣和二尖瓣的打开和关闭时间。输入超声心动图检查前测得的血压。选择“myocardiacwork”程序来显示整体心肌工作的靶心图。获得左心室心肌的总体心肌功指数 （MWI） 参数，其中包括 （1） GCW，代表收缩期心肌缩短或舒张心肌延长所做的积极工作，有助于左心室射血;（2） GWW，代表收缩期心肌延长或舒张期心肌缩短的负功，对抗左心室射血;（3） GWE的计算方式为GCW/（GCW + GWW）;（4） GWI，它是 17 个心肌节段 MWI 的加权平均值（见图 .1）.将血压值输入到 EchoPAC 工作站中，在从主动脉瓣和二尖瓣获得的光谱上标记瓣膜的打开和关闭时间。选择自动功能成像模式，追踪根尖三腔、四腔、两腔动态图像的心内膜边界。根据需要对自动追踪的心内膜轮廓进行手动调整。使用 17 段模型，软件将 GLS 计算为所有段的峰值收缩纵向应变的加权平均值，生成纵向应变曲线和靶心图以供存储。如果区段跟踪的任何一个或多个区域不令人满意，则参与者将被排除在研究之外。然后，打开 Event Timing（事件计时）以确定基于频谱多普勒的主动脉瓣和二尖瓣的打开和关闭时间。输入超声心动图检查前测得的血压。选择“myocardiacwork”程序来显示整体心肌工作的靶心图。获得左心室心肌的总体心肌功指数 （MWI） 参数，其中包括 （1） GCW，代表收缩期心肌缩短或舒张心肌延长所做的积极工作，有助于左心室射血;（2） GWW，代表收缩期心肌延长或舒张期心肌缩短的负功，对抗左心室射血;（3） GWE的计算方式为GCW/（GCW + GWW）;（4） GWI，它是 17 个心肌节段 MWI 的加权平均值（见图 .1）.

### Statistical analysis

Statistical analysis was performed using SPSS version 26.0 (IBM, Armonk, NY) and MedCalc (MedCalc Software bvba, Ostend, Belgium). The distribution of continuous data was evaluated using the Kolmogorov–Smirnov (K-S) test. Normally distributed continuous data were expressed as mean ± standard deviation (*x* ± *s*). Comparisons among three groups were conducted using analysis of variance (ANOVA), and pairwise comparisons between groups were performed using the least significant difference (LSD) *t*-test. For continuous data that did not follow a normal distribution, results were presented as median (interquartile range, *M* (QR)). For multiple group comparisons, the Kruskal–Wallis test was used, and pairwise comparisons were adjusted for significance using the Bonferroni correction. Categorical data were presented as frequency counts and analyzed using the chi-squared (*χ*^2^) test. Spearman correlation analysis was performed to evaluate the correlation between MW parameters and DAS28, GLS, and LVEF. A correlation coefficient of *r* ≥ 0.6 was considered strong; 0.4 ≤ *r* < 0.6 was considered moderate, and *r* < 0.4 was considered weak. A retrospective statistical power analysis was performed using G*Power software (Version 3.1.9.7) to evaluate the adequacy of the sample size for both group comparisons and correlation analyses. For group comparisons, a one-way analysis of variance (ANOVA) was applied. Effect sizes (*f*) were calculated based on the *F*-values, degrees of freedom between and within groups, and the sample size (*N* = 91). Power values were then estimated accordingly. For correlation analyses, Pearson correlation coefficients (*r*) were used to calculate effect sizes (*f*^2^), and power values were determined using a two-tailed test with the sample size (*N* = 62). All analyses assumed a significance level of (*ɑ* = 0.05) and a target power value of (1 − *β* = 0.8).使用 SPSS 26.0 版 （IBM， Armonk， NY） 和 MedCalc （MedCalc Software bvba， Ostend， Belgium） 进行统计分析。使用 Kolmogorov-Smirnov （K-S） 检验评估连续数据的分布。正态分布的连续数据表示为平均值±标准差 （*x* ± *s*）。使用方差分析 （ANOVA） 进行三组间比较，使用最小差异 （LSD） *t* 检验进行组间成对比较。对于不遵循正态分布的连续数据，结果表示为中位数（四分位距，*M* （QR））。对于多组比较，使用 Kruskal-Wallis 检验，并使用 Bonferroni 校正对成对比较进行显着性调整。分类数据以频率计数表示，并使用卡方 （*χ*^2^） 检验进行分析。进行 Spearman 相关性分析以评估 MW 参数与 DAS28 、 GLS 和 LVEF 之间的相关性。相关系数 *r* ≥ 0.6 被认为是强的;0.4 ≤ *r* < 0.6 被认为是中等的，*r* < 0.4 被认为是弱的。使用 G*Power 软件（版本 3.1.9.7）进行回顾性统计功效分析，以评估样本量对组比较和相关分析的充分性。对于组比较，应用单因素方差分析 （ANOVA）。效应量 （*f*） 根据 *F* 计算-值、组间和组内的自由度以及样本量 （*N* = 91）。然后相应地估计功率值。 对于相关性分析，使用 Pearson 相关系数 （*r*） 计算效应量 （*f*^2^），并使用样本量 （*N* = 62） 的双尾检验确定功效值。所有分析均假设显著性水平为 （*ɑ* = 0.05），目标功效值为 （1 − *β* = 0.8）。使用 SPSS 26.0 版 （IBM， Armonk， NY） 和 MedCalc （MedCalc Software bvba， Ostend， Belgium） 进行统计分析。使用 Kolmogorov-Smirnov （K-S） 检验评估连续数据的分布。正态分布的连续数据表示为平均值±标准差 （*x* ± *s*）。使用方差分析 （ANOVA） 进行三组间比较，使用最小差异 （LSD） *t* 检验进行组间成对比较。对于不遵循正态分布的连续数据，结果表示为中位数（四分位距，*M* （QR））。对于多组比较，使用 Kruskal-Wallis 检验，并使用 Bonferroni 校正对成对比较进行显着性调整。分类数据以频率计数表示，并使用卡方 （*χ*^2^） 检验进行分析。进行 Spearman 相关分析以评估 MW 参数与 DAS28 、 GLS 和 LVEF 之间的相关性。相关系数 *r* ≥ 0.6 被认为是强的;0.4 ≤ *r* < 0.6 被认为是中等的，*r* < 0.4 被认为是弱的。使用 G*Power 软件（版本 3.1.9.7）进行回顾性统计功效分析，以评估样本量对组比较和相关分析的充分性。对于组比较，应用单因素方差分析 （ANOVA）。效应量 （*f*） 根据 *F* 计算-值、组间和组内的自由度以及样本量 （*N* = 91）。然后相应地估计功率值。 对于相关性分析，使用 Pearson 相关系数 （*r*） 计算效应量 （*f*^2^），并使用样本量 （*N* = 62） 的双尾检验确定功效值。所有分析均假设显著性水平为 （*ɑ* = 0.05），目标功效值为 （1 − *β* = 0.8）。

### Compliance with ethical standards

This research was approved by the institutional review board of the authors, and informed consent was obtained from all subjects.

## Results

### Comparison of general data among groups

There were no significant differences in age, sex, history of hypertension, diabetes mellitus, smoking history, systolic blood pressure, diastolic blood pressure, and heart rate between the study group and the control group (*p* > 0.05) (Table 1).

**Table 1:** Comparison of general data among groups

Item	Low disease activity	Medium disease activity	High disease activity	Control group	*t*/*Z*	*p*
Number	25	18	19	29	/	/
Male (*n* (%))	5 (27.78%)	6 (24%)	3 (15.79%)	10 (34.48%)	2.169	0.538
Age, years (*M* (P25, P75))	58 (51, 71.5)	63 (53, 71)	62. 5 (53.25, 72.5)	64 (57, 72)	5.973	0.113
HR	73 (68.5, 83)	75 (73, 91)	87.5 (71.7, 96)	74 (67.5, 79.5)	4.984	0.173
Systolic blood pressure	122 (120, 126.5)	120 (115, 125)	122.5 (120, 140)	126 (121, Q143)	7.715	0.052
Diastolic blood pressure	80 (70, 83.5)	70 (75, 80)	81 (73.7, 90)	83 (75, 85)	7.096	0.069
Hypertension (*n* (%))	7 (28%)	9 (50%)	6 (31.58%)	8 (27.59%)	3.039	0.386
Diabetes (*n* (%))	3 (12%)	1 (5.56%)	2 (10.53%)	2 (6.45%)	0.851	0.837
Smoking history (*n* (%))	3 (12%)	3 (16.67%)	1 (5%)	5 (17.24%)	1.824	0.630

### Comparison of disease activity indicators among groups

When comparing the laboratory test indicators (RF, CRP, ESR, NEUT, LYM, PLT) between the study groups and the control group, significant differences were observed (*p* < 0.05). In comparisons between the high disease activity group and the low disease activity group, significant differences were found in ESR, NEUT, and PLT (*p* < 0.05). When compared with the moderate disease activity group, LYM also showed a statistically significant difference (*p* < 0.05). There were statistically significant differences in DAS-28 scores among the three groups with different disease activity levels (*p* < 0.05) (Table 2). 在比较研究组和对照组之间的实验室测试指标（RF、CRP、ESR、NEUT、LYM、PLT）时，观察到显著差异（*p* < 0.05）。在高疾病活动组和低疾病活动组的比较中，发现 ESR、NEUT 和 PLT 存在显着差异 （*p* < 0.05）。与中度疾病活动组相比，LYM 也显示出统计学上的显着差异 （*p*< 0.05）。不同疾病活动水平的三组之间 DAS-28 评分存在统计学显着差异 （*p* < 0.05）（表 2）。

**Table 2 表 2:** Comparison of disease activity indicators among groups组间疾病活动指标比较

Item 项目	Low disease activity 疾病活动度低	Medium disease activity 中等疾病活动度	High disease activity 高疾病活动度	Control group 控制组	*t*/*Z*	*p*
Number 数	25	18	19	29	/	/
RF (IU/mL) 射频 （IU/mL）	66.7 (26.65, 143.50)^a^66.7 （26.65， 143.50）^一个^	266.5 (101.47, 297.75)^a^266.5 （101.47， 297.75）^一个^	214 (39.8, 1256)^a^214 （39.8， 1256） ^一^	11.25 (7.55, 15.47)	66.133	< 0.001
CRP (mg/L) CRP （毫克/升）	22.05 (13.38, 34.42)^a^22.05 （13.38， 34.42）^一个^	23 (16, 37.5)^a^ 23 （16， 37.5）^一个^	71.8 (48, 128.16)^a^71.8 （48， 128.16）^一个^	2.54 (1.8, 9.51)	52.621	< 0.001
ESR (mm/H) ESR （毫米/小时）	41 (23.5, 60.5)^a^41 （23.5， 60.5）^一个^	66.5 (48.7, 78.5)^a^66.5 （48.7， 78.5）^a^	104 (76, 120)^ab^ 104 （76， 120）^AB^	2.5 (2.0, 4.15)	77.323	< 0.001
Neutrophil 中性 粒 细胞	3.82 (2.88, 4.39)	4.73 (3.72, 6.07)^a^4.73 （3.72， 6.07）^一个^	5.38 (3.42, 6.22)^ab^5.38 （3.42， 6.22）^AB^	3.16 (2.18, 3.90)	20.309	< 0.001
Lymphocyte 淋巴细胞	1.52 (1.21, 1.93)	1.65 (1.43, 2.34)^a^1.65 （1.43， 2.34）^一个^	1.32 (0.55, 1.61)^c^1.32 （0.55， 1.61）^摄氏度^	1.46 (1.28, 1.60)	9.984	0.019
Platelet 血小板	243 (212.5, 292)	266 (235.2, 308)	324 (206, 401)^ab^324 （206， 401）^AB^	241 (205, 270)	8.452	0.038
DAS-28 DAS-28 系列	2.84 (2.7, 3.1)	4.28 (3.59, 4.65)^b^4.28 （3.59， 4.65）^乙^	5.9 (5.3, 6.85)^bc^^公元前^ 5.9 （5.3， 6.85）元	/	/	/

*RF* rheumatoid factors, *ESR* erythrocyte sedimentation rate, *CRP* C-reactive protein, *DAS-28* disease activity score*RF* 类风湿因子、*ESR* 红细胞沉降率、*CRP* C 反应蛋白、*DAS-28* 疾病活动度评分

^a^Compared with the control group *p* < 0.05^一个^与对照组相比 *p* < 0.05

^b^Compared with the mild active phase *p* < 0.05^乙^与轻度活性相相比 *p* < 0.05

^c^Compared with the moderately active phase *p* < 0.05^c^与中等活性相相比 *p* < 0.05

### Comparison of conventional echocardiographic parameters, strain parameters, and myocardial work parameters among groups

**Fig. 2:**
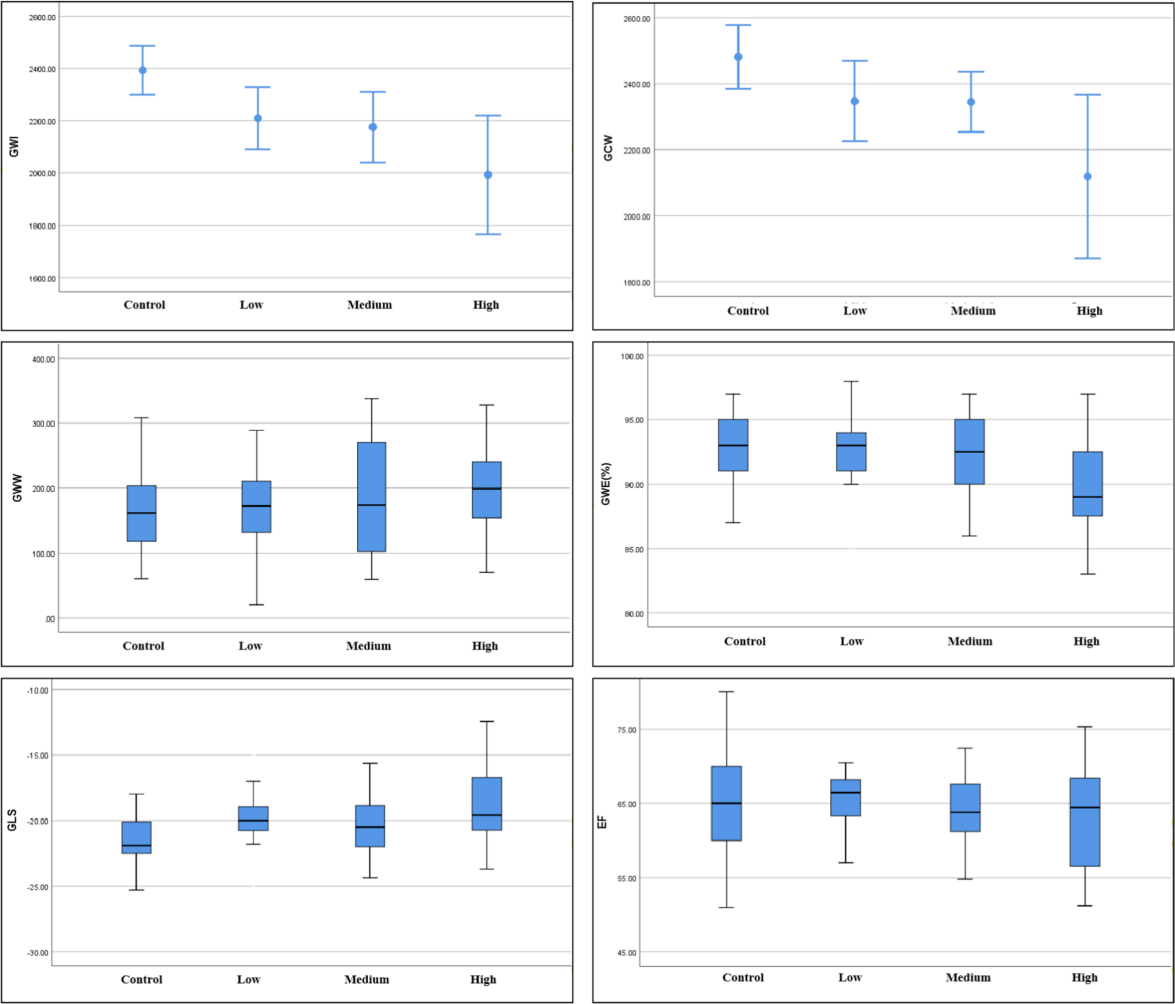
Conventional echocardiographic parameters, strain parameters, and myocardial work parameters among the control, low disease activity, medium disease activity, and high disease activity groups. From the control group to the low, moderate, and high disease activity groups, GWI, GCW, and GWE decreased sequentially. GWI, global work index; GCW, global constructive work; GWW, global wasted work; GWE, global work efficiency; EF, ejection fraction; GLS, global longitudinal strain

No statistically significant differences were found in conventional echocardiographic parameters (EF, IVSD, LVEDV, LVESV, HR) between each study group and the control group (*p* > 0.05). The absolute values of the strain parameter GLS in each case group were significantly lower compared to the control group, with statistical significance (− 19.8 (− 21.1, − 18.7), − 20 (− 22, − 18.86), − 19.85 (− 20.82, − 15.70) vs − 22.2 (− 22.8, − 20) (*p* < 0.05)). From the control group to the low, moderate, and high disease activity groups, GWI, GCW, and GWE decreased sequentially (Fig. 2). The high disease activity group had significantly lower GWI, GCW, and GWE compared to the control group, with statistically significant differences (1992.79 ± 471.39 vs 2421.48 ± 305.70, 2118.63 ± 513.35 vs 2482.22 ± 340.15, 89.5 (87, 92.75) vs 90 (90, 94) (*p* < 0.05)). The GWW of each case group increased sequentially, but the differences were not statistically significant (*p* > 0.05) (Table 3).

**Table 3:** Comparison of conventional echocardiographic parameters, strain parameters, and myocardial work parameters among groups

Item	Low disease activity	Medium disease activity	High disease activity	Control group	*t*/*Z*	*p*
Number	25	18	19	29	/	/
GWI	2209.56 ± 290.44	2175.44 ± 272.18	1992.79 ± 471.39*	2421.48 ± 305.70	8.280	0.001
GCW	2347.20 ± 293.93	2345.06 ± 183.28	2118.63 ± 513.35*	2482.22 ± 340.15	4.955	0.003
GWW	172 (127, 229)	173.5 (102, 271)	199 (150, 246)	161 (118, 203)	2.281	0.536
GWE (%)	92.5 (90.75, 94)	92 (90, 94)	89.5 (87, 92.75)*	90 (90, 94)	9.523	0.023
GLS	− 19.8 (− 21.1, − 18.7)*	− 20 (− 22, − 18.86)*− 20 （− 22， − 18.86）*	− 19.85 (− 20.82, − 15.70)*	− 22.2 (− 22.8, − 20)	17.278	0.001
PSD	50.78 (41.63, 63.25)	44.75 (35.3, 54)	48.27 (39.91, 68.54)	50 (42.05, 63)	5.428	0.134
LVEF (%)	67.3 (63.5, 68.2)	64 (61.2, 67.6)	65.4 (59.9, 70.9)	61 (56, 68.5)	2.370	0.499
IVSD (mm)	7.6 (7.1, 8.8)	7.9 (7.3, 8.4)	8.4 (7.9, 9.2)	8 (7.2, 8.75)	4.432	0.198
LVEDV ()	95.15 (79.28, 111.82)	91.55 (80.08, 104.38)	99.60 (92.2, 126.7)	96.85 (86.03, 113.15)	3.018	0.375
LVESV ()	30.95 (26.35, 39)	33.3 (24.63, 41.15)	43.3 (26.6, 50.3)	32.7 (26.6, 43.3)	3.404	0.333
HR	73 (68.5, 83)	75 (73, 91)	81.5 (71.75, 96)	74 (67.5, 79.5)	4.984	0.173

^*^Compared with the control group *p* < 0.05

### Correlation analysis of myocardial work parameters with GLS, EF, and DAS28心肌工作参数与 GLS 、 EF 和 DAS28 的相关性分析

**Fig. 3:**
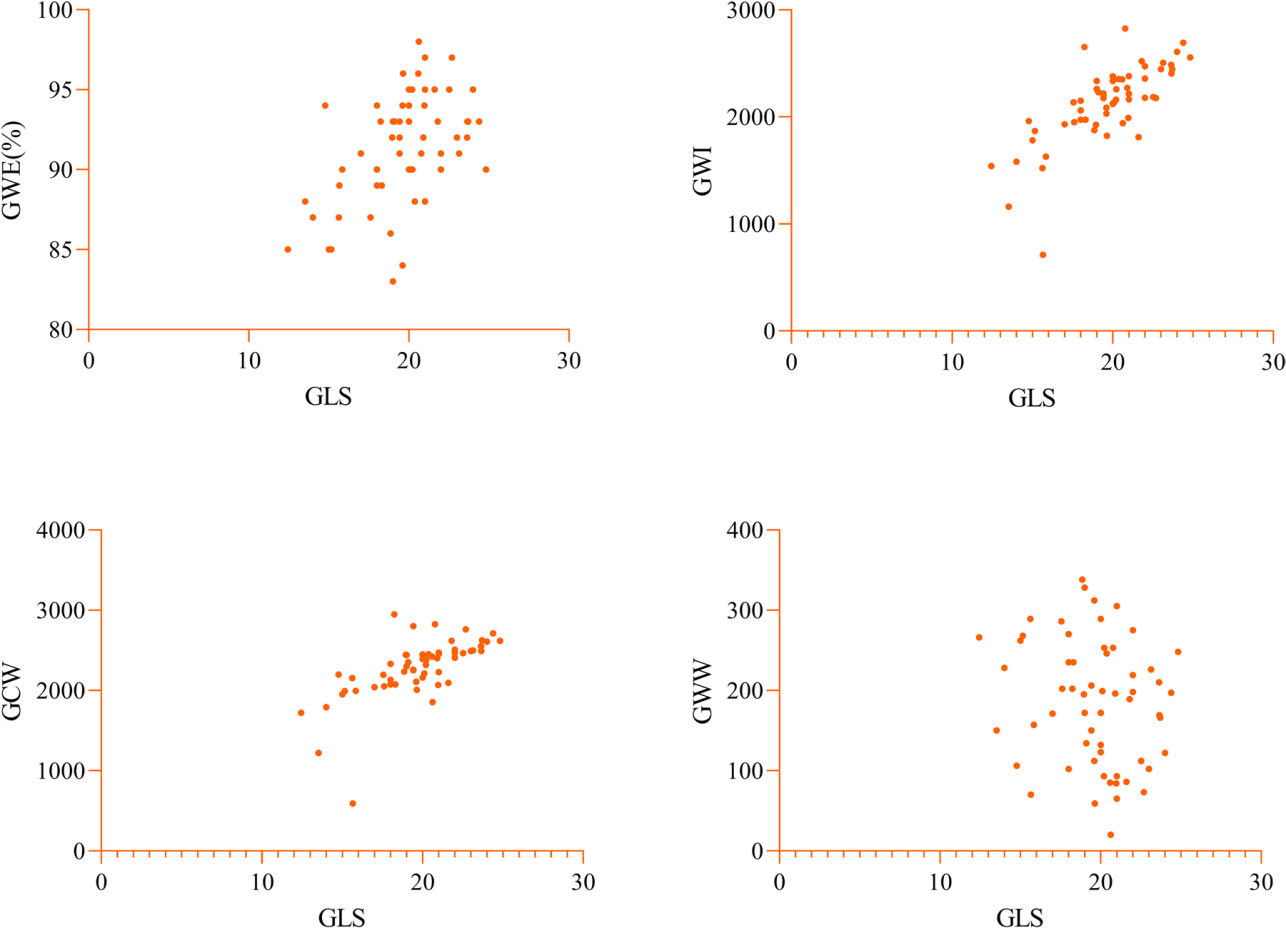
The correlations of myocardial work parameters with GLS in the study groups. GWE, GWI, and GCW in the study group were moderately to strongly positively correlated with the absolute value of GLS, while GWW showed no significant correlation with GLS. GWI, global work index; GCW, global constructive work; GWW, global wasted work; GWE, global work efficiency; GLS, global longitudinal strain研究组中心肌工作参数与 GLS 的相关性。研究组的 GWE 、 GWI 和 GCW 与 GLS 的绝对值呈中度至强正相关，而 GWW 与 GLS 无显著相关性。GWI，全球工作指数;GCW，全球建设性工作;GWW，全球浪费的工作;GWE，全球工作效率;GLS， 整体纵向应变

**Fig. 4:**
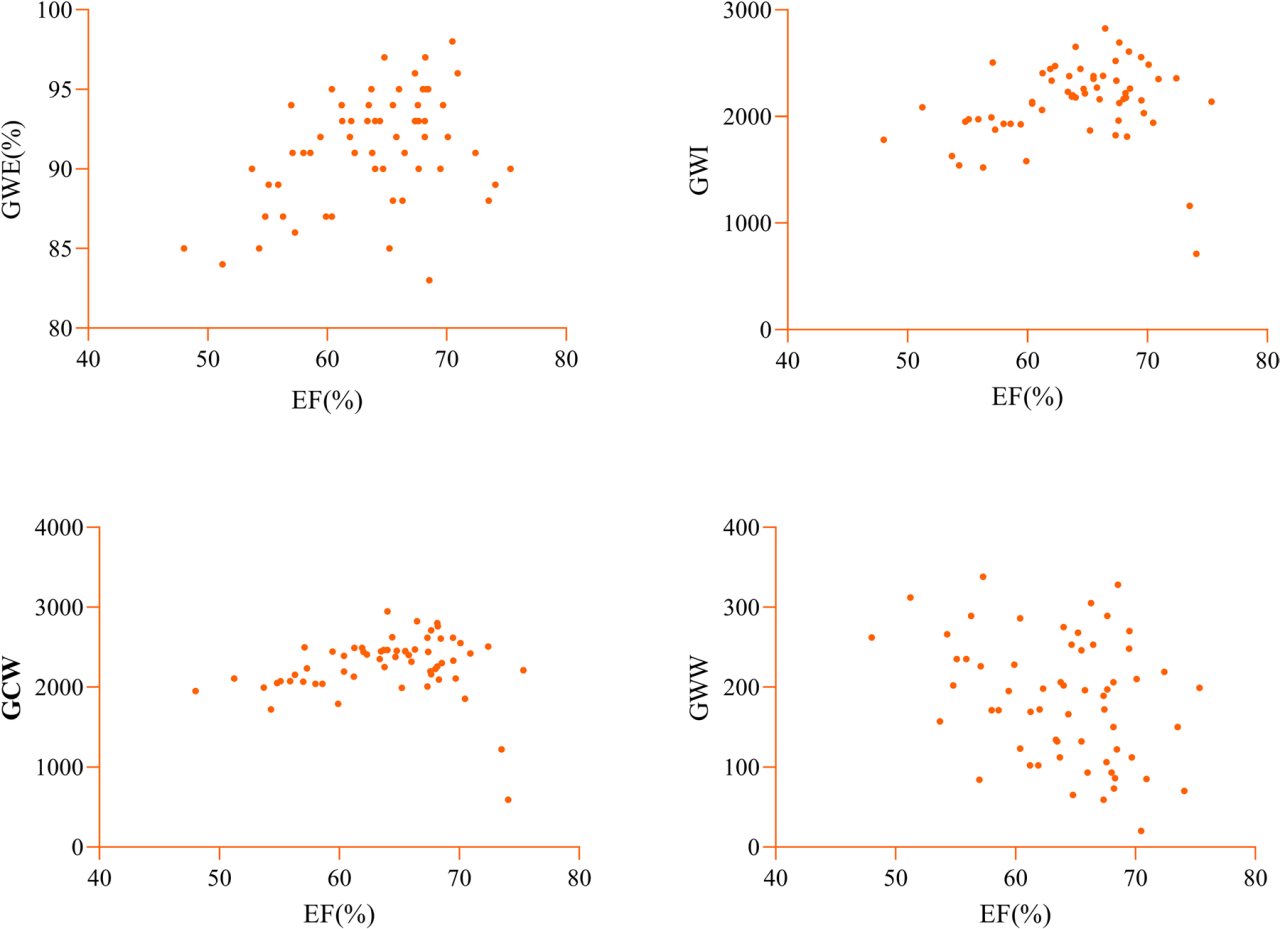
The correlations of myocardial work parameters with EF in the study groups. The MW parameters showed weak correlations with LVEF. GWI, global work index; GCW, global constructive work; GWW, global wasted work; GWE, global work efficiency; EF, ejection fraction

**Fig. 5:**
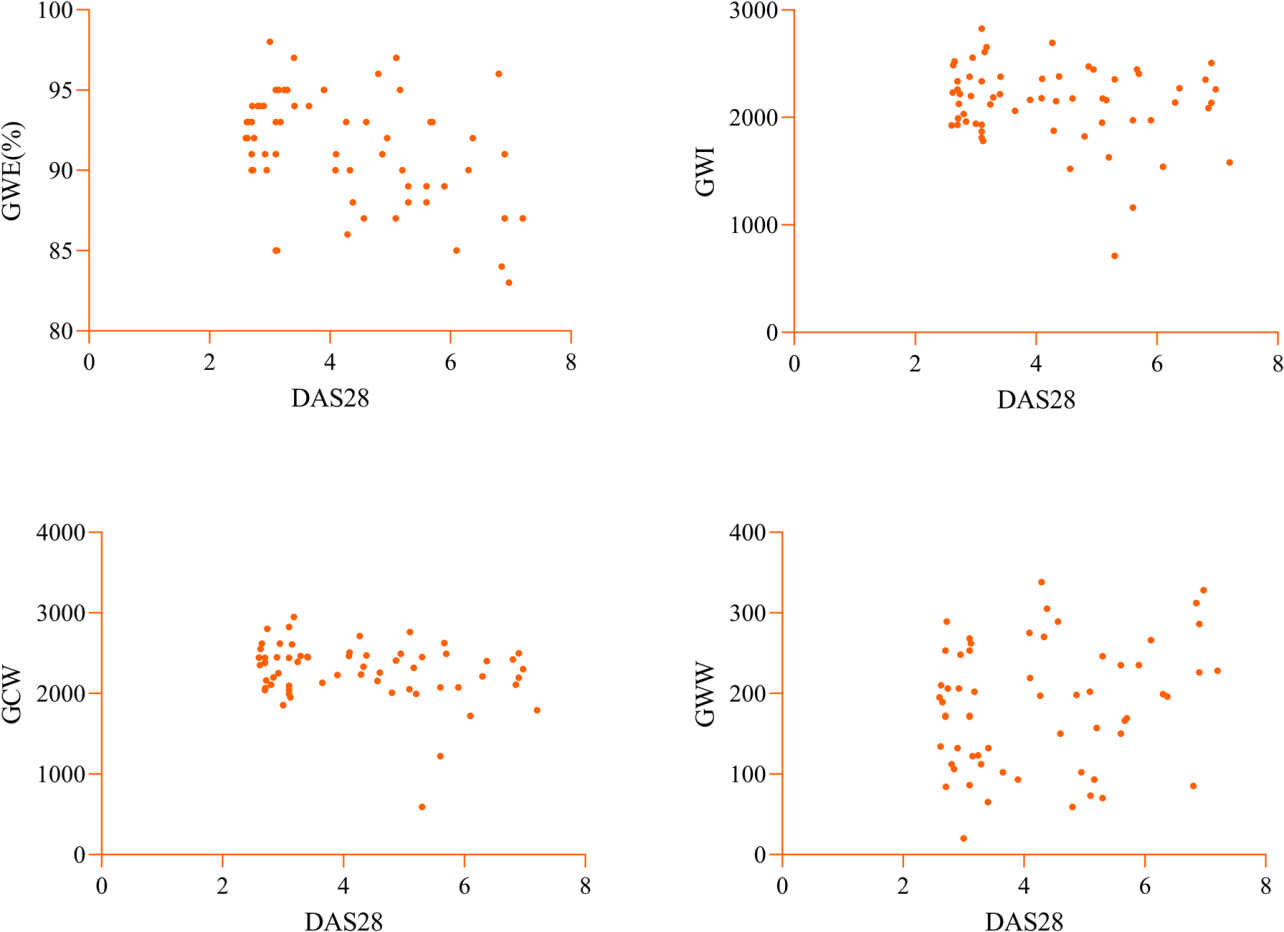
The correlations of myocardial work parameters with DAS28 in the study groups. GWE in the study group exhibited a moderate negative correlation with DAS-28, while other MW parameters showed no significant correlation with DAS-28. GWI, global work index; GCW, global constructive work; GWW, global wasted work; GWE, global work efficiency; DAS28, the 28 joint disease activity score研究组心肌工作参数与 DAS28 的相关性。研究组的 GWE 与 DAS-28 呈中度负相关，而其他 MW 参数与 DAS-28 无显著相关性。GWI，全球工作指数;GCW，全球建设性工作;GWW，全球浪费的工作;GWE，全球工作效率;DAS28,28 关节疾病活动度评分

Spearman correlation analysis was conducted to evaluate the correlations of myocardial work parameters with GLS, EF, and DAS28 in the study group (Table 4). The results indicated that GWE, GWI, and GCW in the study group were moderately to strongly positively correlated with the absolute value of GLS (*r* = − 0.501, − 0.746, − 0.666; all *p* < 0.01), while GWW showed no significant correlation with GLS (*p* > 0.05) (Fig. 3). The MW parameters showed weak correlations with LVEF (*p* < 0.05) (Fig. 4). GWE in the study group exhibited a moderate negative correlation with DAS-28 (*r* = − 0.402, *p* < 0.05), while other MW parameters showed no significant correlation with DAS-28 (all *p* > 0.05) (Fig. 5).

**Table 4:** Correlation analysis of myocardial work parameters with GLS, EF, and DAS28

	GLS		EF		DAS28	
	*r*	*p*	*r*	*p*	*r*	*p*
GWE	0.501	< 0.001	0.331	0.009	− 0.402	0.001
GWI	0.746	< 0.001	0.270	0.034	− 0.197	0.124
GCW	0.666	< 0.001	0.254	0.046	− 0.243	0.057
GWW	− 0.175	0.173	− 0.265	0.037	0.027	0.076

### Statistical power analysis results

The statistical power analysis demonstrated that the sample sizes for both group comparisons and correlation analyses were sufficient to ensure reliable findings.

For group comparisons (*N* = 91), myocardial parameters such as GWI, GCW, GWE, and GLS showed effect sizes of *f* = 0.535, *f* = 0.413, *f* = 0.573, and* f* = 0.771, respectively, with corresponding power values of 0.961, 0.956, 0.962, and 0.967. These results indicate a high level of statistical reliability for the observed group differences.对于组比较 （*N* = 91），GWI、GCW、GWE 和 GLS 等心肌参数显示效应量分别为 *f* = 0.535、*f* = 0.413、*f* = 0.573 和* f* = 0.771，相应的功效值为 0.961、0.956、0.962 和 0.967。这些结果表明，观察到的组差异具有高水平的统计可靠性。

For correlation analyses (*N* = 62), strong correlations were observed between GWI and GLS (*r* = 0.746) and between GWE and GLS (*r* = 0.501), with power values exceeding 0.999 and 0.998, respectively, confirming the robustness of these relationships. Additionally, the correlation between GWE and DAS28 (*r* = − 0.402) demonstrated a moderate negative relationship with a power value of 0.907.

## Discussion

According to the data released by the Global Burden of Disease Study in 2021, the global mortality rate from rheumatoid arthritis (RA) has decreased over the past 30 years, showing a reduction of 23.8% (17.5–29.3) from 1990 to 2020. A nationwide population-based matched cohort study from Denmark reported similar findings [10]. However, the prevalence of RA has increased by 14.1% (12.7–15.4). In 2020, an estimated 17.6 million (95% uncertainty interval 15.8–20.3) people worldwide were living with rheumatoid arthritis. The incidence of RA in China has significantly increased over the past 30 years. From 1990 to 2021, the age-standardized incidence rates (ASIR) of RA in China increased from 11.6 to 13.7 [11]. The number of cases is expected to continue rising by 2050, highlighting the need for improvements in early diagnosis and treatment of RA globally to alleviate its future burden [12].

The pathogenesis of RA remains unclear [13], but cardiovascular events are the leading cause of death among RA patients, with a significant portion attributed to atherosclerotic diseases [14]. The increased risk of cardiovascular events in RA is caused by a complex interaction between traditional risk factors and disease-related risk factors, with chronic inflammation and persistent disease activity being key determinants of this risk [15]. Long-term chronic inflammation and the body’s autoimmune response lead to damage and repair related to immune system dysfunction [16]. Inflammation plays a role in accelerating vascular remodeling and the formation of atherosclerosis in autoimmune diseases like RA [17, 18]. Strict control of inflammation is critical in reducing cardiovascular risk [19, 20]. Additionally, clinical treatments for RA patients often include steroids and non-steroidal anti-inflammatory drugs (NSAIDs), which may increase cardiovascular risk due to their side effects.

The early symptoms and signs of cardiac damage in RA are often subtle. Clinically, assessments of cardiac function can be performed using myocardial enzyme profiles and electrocardiograms (ECGs); however, the sensitivity of these tests is low, and they require strict timing for onset, limiting their application. While endomyocardial biopsy can serve as a gold standard for detecting myocardial damage, it is invasive and therefore not suitable for routine screening. Magnetic resonance imaging (MRI) and radionuclide ventriculography can also evaluate cardiac function but are cost-prohibitive, restricting their clinical use. Consequently, the diagnostic rate for early myocardial dysfunction in RA is relatively low.RA 心脏损伤的早期症状和体征通常很微妙。临床上，可以使用心肌酶谱和心电图 （ECG） 进行心脏功能评估;然而，这些检查的敏感性较低，并且它们需要严格的发病时间，从而限制了它们的应用。虽然心内膜心肌活检可以作为检测心肌损伤的金标准，但它是侵入性的，因此不适合常规筛查。磁共振成像 （MRI） 和放射性核素脑室造影也可以评估心脏功能，但成本高昂，限制了它们的临床应用。因此，RA 早期心肌功能障碍的诊断率相对较低。

Our study also suggests that traditional echocardiographic parameters have limited sensitivity in detecting early cardiac damage in RA. The results indicate that there are statistically significant differences in laboratory test indicators (RF, CRP, ESR, NEUT, LYM, PLT) when comparing the case groups with the control group (*p* < 0.05), indicating a severe inflammatory response in active RA patients. Furthermore, significant differences in ESR, NEUT, and PLT were found between the high disease activity group and the low disease activity group (*p* < 0.05). Studies have shown that long-term immune responses and chronic inflammation in RA patients accelerate myocardial cell apoptosis, directly damaging myocardial tissue [21, 22]. Inflammatory and immune cells release various pro-fibrotic factors during the inflammatory response, increasing fibroblast activity and causing interstitial fibrosis in the myocardium, which, in turn, promotes atherosclerosis, leading to myocardial ischemia and impaired left ventricular systolic and diastolic function [23]. However, all conventional echocardiographic parameters in our study’s subjects were within the normal range, and there were no statistically significant differences in conventional echocardiographic results (EF, IVSD, LVEDV, LVESV, HR) between the case and control groups (*p* > 0.01). This indicates that the above conventional echocardiographic parameters have certain limitations in evaluating cardiac function impairment in RA patients.

The pressure-strain loop (PSL) technique combines myocardial strain data obtained from echocardiography with non-invasive estimates of left ventricular pressure to create pressure-strain loop diagrams. The LV-PSL considers the impact of afterload on strain, overcoming the load dependency of strain data obtained through two-dimensional speckle-tracking technology, allowing for a more accurate assessment of left ventricular myocardial function impairment [24]. Previous studies have reported that echocardiographic PSL results for assessing myocardial work are significantly correlated with cardiac catheterization results [25]. Chan et al. [26] applied PSL to assess myocardial work in patients with three different cardiovascular conditions. Their research showed that hypertensive patients with systolic blood pressure > 160 mmHg had significantly increased left ventricular GWI and GCW compared to healthy controls, while GLS and LVEF remained normal and relatively unchanged. The development of the PSL technique provides a more comprehensive tool for assessing myocardial function; it can quantify cardiac motion as well as assess cardiac work efficiency and has a clear relationship with myocardial metabolism [27]. As a non-invasive examination method, PSL technology has seen wide-ranging applications and developments in the field of cardiology. A study divided patients with hyperthyroidism into two groups: the tachycardia group (TH1, *n* = 31) and the non-tachycardia group (TH2, *n* = 34). They used non-invasive PSL technology to evaluate left ventricular function. The results indicated that hyperthyroidism significantly reduced the GWE of the left ventricle and increased GWW, with these changes being more pronounced in patients with tachycardia [28]. Another study evaluated asymptomatic left ventricular myocardial systolic dysfunction in patients with type 2 diabetes mellitus (T2DM) with and without hypertension (HT) through PSL technology. The findings revealed that GWI, GCW, and the ratios of GWE and GWW were significantly lower in T2DM patients with hypertension compared to normal controls (*p* < 0.05). Furthermore, the LV anterior wall work index (WI), active work (CW), work efficiency (WE), and the CW/WW ratio in both the T2DM group and the T2DM with HT group were noticeably lower than those in the normal control group (*p* < 0.05) [29]. This evidence demonstrates that myocardial work can be assessed non-invasively and accurately in identifying asymptomatic global and localized LV contractile dysfunction in T2DM patients with or without hypertension. The advent of PSL technology has deepened our understanding of myocardial function in patients, serving as a promising new tool for the early detection of subclinical left ventricular dysfunction.压力-应变环 （PSL） 技术将从超声心动图获得的心肌应变数据与左心室压力的无创估计相结合，以创建压力-应变环图。LV-PSL 考虑了后负荷对应变的影响，克服了通过二维斑点跟踪技术获得的应变数据的负荷依赖性，从而可以更准确地评估左心室心肌功能损害 []。既往研究报道，用于评估心肌工作的超声心动图 PSL 结果与心导管检查结果显著相关 []。Chan等[]应用PSL来评估三种不同心血管疾病患者的心肌工作。他们的研究表明，与健康对照组相比，收缩压> 160 mmHg 的高血压患者的左心室 GWI 和 GCW 显着增加，而 GLS 和 LVEF 保持正常且相对不变。PSL 技术的发展为评估心肌功能提供了更全面的工具;它可以量化心脏运动以及评估心脏工作效率，并且与心肌代谢有明确的关系 []。作为一种无创检查方法，PSL 技术在心脏病学领域得到了广泛的应用和发展。一项研究将甲状腺功能亢进症患者分为两组：心动过速组 （TH1， *n* = 31） 和非心动过速组 （TH2， *n* = 34）。他们使用无创 PSL 技术来评估左心室功能。 结果表明，甲状腺功能亢进症显著降低了左心室的 GWE，增加了 GWW，这些变化在心动过速患者中更为明显 []。另一项研究通过 PSL 技术评估了 2 型糖尿病 （T2DM） 伴和不伴高血压 （HT） 患者的无症状左心室心肌收缩功能障碍。研究结果显示，与正常对照相比，T2DM 高血压患者的 GWI、GCW 以及 GWE 和 GWW 的比率显着降低 （*p* < 0.05）。此外，T2DM 组和 T2DM 伴 HT 组的 LV 前壁工作指数 （WI） 、主动功 （CW） 、工作效率 （WE） 和 CW/WW 比率均明显低于正常对照组 （*p* < 0.05） []。该证据表明，在识别伴有或不伴有高血压的 T2DM 患者的无症状整体和局部 LV 收缩功能障碍方面，可以无创准确地评估心肌工作。PSL 技术的出现加深了我们对患者心肌功能的理解，成为早期发现亚临床左心室功能障碍的有前途的新工具。

GLS is considered a sensitive indicator for assessing early changes in cardiac function [30]. Yasser Gazar et al. [31] applied speckle-tracking echocardiography (STE) strain technology to evaluate left ventricular systolic function in rheumatoid arthritis (RA) patients without cardiovascular diseases. They found that GLS in the RA group was reduced (− 16.80% vs − 22.35%, *p* < 0.001). Using receiver operating characteristic (ROC) curve analysis, the optimal cutoff value for GLS was determined to be − 20, with a sensitivity of 76.7%, specificity of 80%, positive predictive value of 92%, negative predictive value of 63%, and an overall diagnostic accuracy of 83.9%. Thus, GLS measurements using STE are valuable for detecting left ventricular systolic dysfunction in RA patients with preserved ejection fraction. A study by Giovanni Cioffi et al. [32] found that low GLS and low global circumferential strain (GCS) are strong independent predictors of cardiovascular events in RA patients. In this study, the absolute values of strain parameters (GLS) in each case group were significantly reduced compared to the control group (*p* < 0.05). This indicates that GLS can sensitively detect subclinical left ventricular dysfunction in RA patients, potentially related to the anatomical structure of the left ventricular myocardium. The left ventricular myocardium consists of longitudinal, oblique, and circumferential fibers. Myocardial longitudinal strain is primarily influenced by the longitudinal muscle fibers, which are located subendocardially and are typically the first to be affected during myocardial disease. Correlation analysis in this study showed that GWI and GCW were significantly related to GLS, consistent with previous studies [33]. Therefore, PSL technology is feasible in assisting with the assessment of early myocardial function in RA patients. However, this study found that the absolute value of GLS in the high disease activity group was lower than that in the low disease activity group, but the difference was not statistically significant. This may be related to the fact that GLS measurements are easily affected by afterload [34, 35]. Thus, although GLS is very sensitive for assessing early myocardial function impairment, the impact of afterload cannot be avoided. Combining the latest PSL technology can provide more accurate and comprehensive results.

Our study on myocardial work showed a sequential decrease in GWI, GCW, and GWE among the control group, low disease activity group, moderate disease activity group, and high disease activity group, with significant reductions in GWI, GCW, and GWE in the high disease activity group compared to the control group. This indicates that myocardial work capacity in RA patients decreases with escalating disease activity. Correlation analysis revealed that GWE was moderately correlated with both GLS and DAS-28, suggesting a close relationship between RA disease activity and myocardial dysfunction. In recent years, increasing research attention has been paid to the relationship between disease activity in RA and myocardial damage. As early as 2013, Nowell M. Fine et al. [36] found that both left ventricular and right ventricular longitudinal strain were reduced in RA patients compared to healthy subjects, and strain abnormalities were associated with the severity of RA. Małgorzata Biskup et al. [37] found that sustained low disease activity of RA was associated with atherosclerosis and cardiac dysfunction. A 5-year prospective study found that patients with sustained moderate or high disease activity significantly increased the risk of subclinical atherosclerosis progression (AP +) compared to those who achieved sustained remission (OR 5.05, 95% CI 1.53–16.64, *p* = 0.008) [38]. Punchong Hanvivadhanakul’s research [39] found that RA patients without clinical cardiovascular disease showed reduced left ventricular systolic function, characterized by lower GLS, which was significantly correlated with disease activity (DAS28-CRP). These findings align with our research and underscore the importance of incorporating advanced echocardiographic techniques into routine cardiovascular risk assessment for RA patients, especially those with high disease activity. The underlying mechanism may involve the deposition of immune complexes caused by inflammatory responses in RA patients within the vascular walls, affecting small branches of the coronary arteries and accelerating atherosclerosis in the coronary microvasculature. This leads to reduced perfusion in the peripheral circulation, ultimately causing narrowing of the coronary lumen and resulting in ischemia of the subendocardial myocardium, which is primarily supplied by peripheral blood vessels [40]. Subendocardial longitudinal myocardial fibers play a particularly important role in the motion of the left ventricle’s long axis. Studies have indicated that if longitudinal myocardial fibers do not participate in ventricular contraction, the ejection fraction generated from myocardial segment shortening will significantly decline, and the contraction of subendocardial longitudinal myocardial fibers is more susceptible to the effects of myocardial ischemia [41]. Additionally, inflammatory and cytotoxic factors (including anti-lipid antibodies, anti-citrullinated protein antibodies, coagulation factors, and tissue plasminogen activator antigens) can also lead to myocardial fibrosis and apoptosis. The combined effects of these factors will accelerate myocardial injury [21, 42]. As the inflammation intensifies and the condition of RA patients progresses, the myocardial strain capacity decreases, leading to a decline in myocardial function.我们对心肌工作的研究表明，对照组、低疾病活动组、中度疾病活动组和高疾病活动组的 GWI 、 GCW 和 GWE 依次降低，与对照组相比，高疾病活动组的 GWI 、 GCW 和 GWE 显著降低。这表明 RA 患者的心肌工作能力随着疾病活动度的升高而降低。相关性分析显示，GWE 与 GLS 和 DAS-28 均呈中度相关性，表明 RA 疾病活动与心肌功能障碍之间存在密切关系。近年来，RA 疾病活动与心肌损伤之间的关系越来越受到研究关注。早在 2013 年，Nowell M. Fine 等 [] 就发现，与健康受试者相比，RA 患者的左心室和右心室纵向应变均有所降低，并且应变异常与 RA 的严重程度相关。Małgorzata Biskup等[]发现RA的持续低疾病活动度与动脉粥样硬化和心功能不全有关。一项为期 5 年的前瞻性研究发现，与达到持续缓解的患者相比，持续中度或高度疾病活动的患者亚临床动脉粥样硬化进展 （AP +） 的风险显著增加（OR 5.05,95% CI 1.53-16.64，p = 0.008 ）[]。Punchong Hanvivadhanakul 的研究 [] 发现，无临床心血管疾病的 RA 患者表现为左心室收缩功能降低，其特征是 GLS 降低，这与疾病活动度 （DAS28-CRP） 显著相关。 这些发现与我们的研究一致，并强调了将先进的超声心动图技术纳入 RA 患者（尤其是疾病活动度高的患者）的常规心血管风险评估的重要性。潜在机制可能涉及 RA 患者炎症反应引起的免疫复合物在血管壁内的沉积，影响冠状动脉的小分支并加速冠状动脉微血管系统中的动脉粥样硬化。这导致外周循环灌注减少，最终导致冠状动脉腔狭窄，并导致心内膜下心肌缺血，而心内膜下心肌主要由外周血管供血[]。心内膜下纵向心肌纤维在左心室长轴的运动中起着特别重要的作用。研究表明，如果纵向心肌纤维不参与心室收缩，心肌段缩短产生的射血分数会明显下降，心内膜下纵向心肌纤维的收缩更容易受到心肌缺血的影响[].此外，炎症和细胞毒因子（包括抗脂质抗体、抗瓜氨酸蛋白抗体、凝血因子和组织纤溶酶原激活剂抗原）也会导致心肌纤维化和细胞凋亡。这些因素的综合影响会加速心肌损伤 [， ]。随着炎症的加剧和 RA 患者病情的进展，心肌应变能力下降，导致心肌功能下降。

RA patients are known to have an elevated cardiovascular risk, although the underlying mechanisms remain incompletely understood. Our results suggest that myocardial work parameters, particularly GWE, may serve as novel biomarkers for subclinical cardiovascular dysfunction in RA. The significant correlation observed between GWE and disease activity (DAS28) highlights the interplay between systemic inflammation and myocardial performance. GWE and other myocardial work parameters may help identify subgroups of RA patients at higher cardiovascular risk who could benefit from earlier and more intensive intervention, such as tailored anti-inflammatory therapy or closer cardiovascular monitoring.已知 RA 患者的心血管风险升高，但其潜在机制仍不完全清楚。我们的结果表明，心肌工作参数，尤其是 GWE，可能作为 RA 亚临床心血管功能障碍的新型生物标志物。在 GWE 和疾病活动 （DAS28） 之间观察到的显著相关性突出了全身炎症与心肌表现之间的相互作用。GWE 和其他心肌工作参数可能有助于确定心血管风险较高的 RA 患者亚组，这些患者可以从更早和更深入的干预中受益，例如量身定制的抗炎治疗或更密切的心血管监测。

In conclusion, PSL technology can identify potential myocardial dysfunction in RA patients, even with normal LVEF. These subtle functional changes may be overlooked by traditional cardiac function assessment methods, but they hold significant clinical implications for the early evaluation and cardiovascular risk stratification in patients with RA, allowing for timely treatment planning. This study demonstrates that ultrasonic echocardiography using PSL technology to detect myocardial workload parameters can serve as a non-invasive tool to assist in the early diagnosis of myocardial dysfunction in RA patients. It provides a non-invasive, convenient, economical, and reliable detection method for the early identification of myocardial dysfunction in RA patients, guiding treatment strategies and predicting patient prognosis.总之，PSL 技术可以识别 RA 患者的潜在心肌功能障碍，即使 LVEF 正常。这些细微的功能变化可能会被传统的心脏功能评估方法所忽视，但它们对 RA 患者的早期评估和心血管风险分层具有重要的临床意义，从而可以及时制定治疗计划。这项研究表明，使用 PSL 技术检测心肌负荷参数的超声超声心动图可以作为一种无创工具，辅助 RA 患者心肌功能障碍的早期诊断。它为 RA 患者心肌功能障碍的早期识别、指导治疗策略和预测患者预后提供了一种无创、便捷、经济、可靠的检测方法。

The statistical power analysis confirmed that the sample sizes in this study were sufficient to ensure the reliability and robustness of the findings in both group comparisons and correlation analyses. For group comparisons (*N* = 91), myocardial parameters (GWI, GCW, GWE, and GLS) demonstrated medium-to-large effect sizes, with power values significantly exceeding 0.8, underscoring the high reliability of the observed group differences.统计功效分析证实，本研究中的样本量足以确保组比较和相关分析结果的可靠性和稳健性。对于组比较 （*N* = 91），心肌参数 （GWI、GCW、GWE 和 GLS） 显示中等到大的效应量，功效值显著超过 0.8，强调了观察到的组差异的高可靠性。

For correlation analyses (*N* = 62), the strong relationships between GWI and GLS (*r* = 0.746) and between GWE and GLS (*r* = 0.501) were supported by near-maximal power values (> 0.999 and 0.998, respectively). These results further validate the significance of these associations. However, for the correlation between GWE and DAS28 (*r* = − 0.402), although the power value was 0.907, the relatively small effect size and limited sample size indicate that future studies with larger sample sizes are needed to confirm the robustness and generalizability of weaker correlations.对于相关性分析 （*N* = 62），GWI 和 GLS （*r* = 0.746） 以及 GWE 和 GLS 之间的强关系 （*r* = 0.501） 得到了近最大功效值 （分别为 >0.999 和 0.998） 的支持。这些结果进一步验证了这些关联的重要性。然而，对于 GWE 和 DAS28 之间的相关性 （*r* = − 0.402），虽然功效值为 0.907，但相对较小的效应量和有限的样本量表明，未来需要更大样本量的研究来证实较弱相关性的稳健性和普遍性。

Although the statistical power analysis provides strong support for the credibility of the observed group differences and relationships in this study, these results also offer valuable guidance for future studies in terms of sample size planning and effect size estimation. This study has certain limitations that warrant consideration. First, the relatively small sample size (*n* = 62) may limit the statistical power for detecting smaller effect sizes and could reduce the ability to conduct subgroup analyses, particularly for certain cardiovascular risk factors in RA patients. Furthermore, as a single-center study, the generalizability of our findings may be limited due to potential selection bias inherent to the specific cohort. Future research should include larger, multicenter cohorts to confirm our findings in more diverse patient populations, enhance statistical robustness, and explore subgroup-specific effects. We also encourage prospective studies to examine the longitudinal implications of myocardial work parameters and their role in stratifying cardiovascular risk in RA patients.尽管统计功效分析为本研究中观察到的组差异和关系的可信度提供了有力支持，但 these 结果也为未来的研究在样本量规划和效应量估计方面提供了有价值的指导。这项研究有一定的局限性，值得考虑。首先，相对较小的样本量 （*n* = 62） 可能会限制检测较小效应量的统计能力，并可能降低进行亚组分析的能力，特别是对于 RA 患者的某些心血管危险因素。此外，作为一项单中心研究，由于特定队列固有的潜在选择偏倚，我们研究结果的普遍性可能会受到限制。未来的研究应包括更大的多中心队列，以证实我们在更多样化的患者群体中的发现，增强统计稳健性，并探索亚组特异性效应。我们还鼓励前瞻性研究来检查心肌工作参数的纵向影响及其在 RA 患者心血管风险分层中的作用。

## Data Availability

Data will be made available on request.
